# Identification of Genes Relevant to Pesticides and Biology from Global Transcriptome Data of *Monochamus alternatus* Hope (Coleoptera: Cerambycidae) Larvae

**DOI:** 10.1371/journal.pone.0147855

**Published:** 2016-01-27

**Authors:** Songqing Wu, Xiaoli Zhu, Zhaoxia Liu, Ensi Shao, Carballar-Lejarazú Rebeca, Yajie Guo, Yueting Xiong, Yani Mou, Runxue Xu, Xia Hu, Guanghong Liang, Shuangquan Zou, Xiong Guan, Feiping Zhang

**Affiliations:** 1 College of Forestry, Fujian Agriculture and Forestry University, Fuzhou, 350002, People’s Republic of China; 2 Fujian-Taiwan Joint Center for Ecological Control of Crop Pests, Fujian Agriculture and Forestry University, Fuzhou, 350002, People’s Republic of China; 3 Key Laboratory of Biopesticide and Chemical Biology, Ministry of Education, Fujian Agriculture and Forestry University, Fuzhou, 350002, People’s Republic of China; 4 Department of Molecular Biology and Biochemistry, University of California Irvine, Irvine, CA, 92697, United States of America; Institute of Hydrobiology, Chinese Academy of Sciences, CHINA

## Abstract

*Monochamus alternatus* Hope is the main vector in China of the Pine Wilt Disease caused by the pine wood nematode *Bursaphelenchus xylophilus*. Although chemical control is traditionally used to prevent pine wilt disease, new strategies based in biological control are promising ways for the management of the disease. However, there is no deep sequence analysis of *Monochamus alternatus* Hope that describes the transcriptome and no information is available about gene function of this insect vector. We used next generation sequencing technology to sequence the whole fourth instar larva transcriptome of *Monochamus alternatus* Hope and successfully built a *Monochamus alternatus* Hope transcriptome database. In total, 105,612 unigenes were assigned for Gene Ontology (GO) terms, information for 16,730 classified unigenes was obtained in the Clusters of Orthologous Groups (COGs) database, and 13,024 unigenes matched with 224 predicted pathways in the Kyoto Encyclopedia of Genes and Genome (KEGG). In addition, genes related to putative insecticide resistance-related genes, RNAi, the Bt receptor, intestinal digestive enzymes, possible future insect control targets and immune-related molecules are described. This study provides valuable basic information that can be used as a gateway to develop new molecular tools for *Monochamus alternatus* Hope control strategies.

## Introduction

Pine Wilt Disease is a devastating disease in pine trees caused by the infection of *Bursaphelenchus xylophilus* and it is commonly known as the cancer of pine trees [1]. Since the discovery of *B*. *xylophilus* in Japanese black pines in the Sun Yat-sen Mausoleum in Nanjing City (Jiangsu Province in China) in 1982, Pine Wilt Disease has occurred in a total of 275 county-level administrative regions (excluding Hong Kong and Taiwan) of 17 provinces (autonomous regions and municipalities), causing immense damage to forest resources and having impact in China’s ecological environment [2]. In China, the principal vector for Pine Wilt Disease is the beetle *Monochamus alternatus* Hope (*M*. *alternatus*) larvae as carriers; after emergence, t larvae use pine trees as food and oviposition sources, therefore they are considered the invasive stage of the insect. Effective control of *M*. *alternatus* plays an important role in the prophylaxis and treatment of this disease [3].

At present, the principal strategies to control *M*. *alternatus* include: trap trees, biological control, silvicultural control and chemical prevention [3, 4]. Among these, the biological control presents unique advantages: (1) It is difficult for pests to become resistant as microorganisms have adapted to the immune systems of insects during the process of concurrent evolution. Therefore insect immunity to pathogenic microorganisms has been kept at a low level; (2) It has high environmental security; microorganisms typically have strong specificity for their targets and they are harmless to vertebrates, which protects the natural predators of those hosts; (3) insects are an ideal medium for various types of pathogens; the proliferation of insect pathogens *in vivo* can be spread by diseases and pests or the insect’s body; (4) It’s easy to obtain strains that are strongly pathogenic using genetic engineering and transformation techniques [5, 6]. Current biological control techniques for *M*. *alternatus* have progressed, including the spreading of effective natural enemies, creation of black lights and trap-trees. Application of the above techniques has successfully controlled Pine Wilt Disease at test locations [5]. Among the methods of natural enemies are the parasitoid beetles *Dastarcus helophoroides* and *Sclerodermus* spp. (Hymenoptera: Bethylidae), showing up to 90% of effectiveness [7, 8]. In general, biological control has brought new insights to control *M*. *alternatus* forest infestations. Therefore, microbial control of *M*. *alternatus* has increasingly gained attention [4]. However, there is currently a lack of knowledge regarding *Monochamus alternatus* Hope transcripts, gene expression and gene function in this insect vector.

We used next generation sequencing technology to sequence the whole fourth instar larvae transcriptome of *M*. *alternatus* and successfully built a *M*. *alternatus* Hope transcriptome database. In addition, our data describe genes related to putative insecticide resistance, intestinal digestive enzymes, possible future insect control targets and immune-related molecules. This study provides valuable basic information that can be used as a key point to develop new molecular tools for *M*. *alternatus* Hope control strategies.

## Results and Discussion

### Sequencing and de novo assembly

Illumina sequencing produced 46, 761 and 743 clean reads for the larvae samples, which is equivalent to an accumulated length of 11, 777, 133 and 130 bp (Table 1).

**Table 1:** Sequence statistics of the Illumina sequencing assembly.

	Reads	Contig	Transcript	Unigene
Number of sequences	94,545,777	11,433,166	107,259	73,090
Mean length (bp)	252	42	942	693
Total length (bp)	23,805,690,981	475,935,825	101,013,380	50,668,837

**Fig 1:**
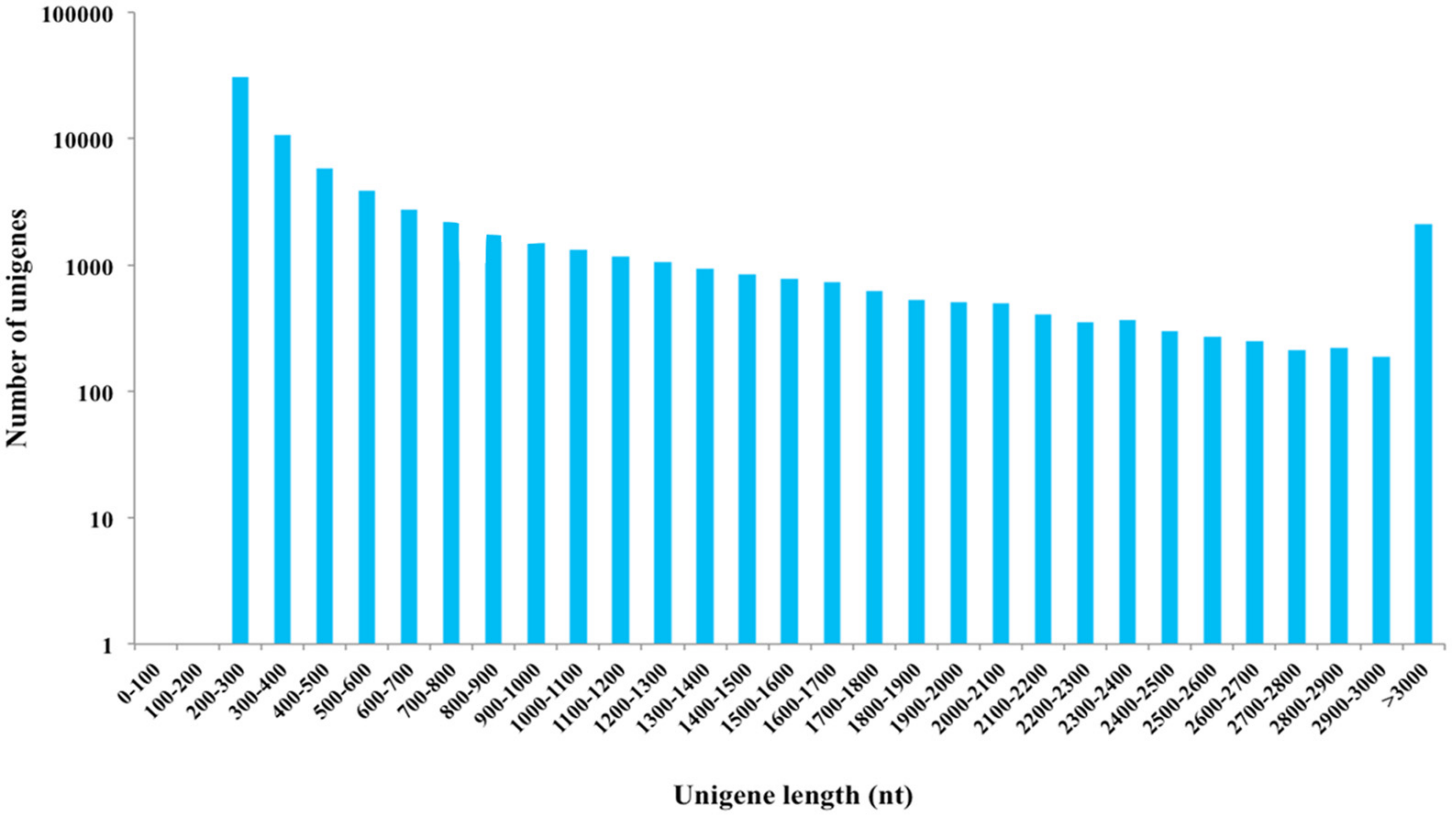
Unigenes length distribution. The y-axis number has been converted into logarithmic scale.

The average of raw reads length was 252 bp. First, the sequencing reads were broken into K-mers using Trinity software [9]; the small fragments were assembled into 11,433,166 contigs with a mean length of 41.63 bp. The length of the contigs mainly ranged from 0 to 3000 bp, representing 99.61% of the reads, although some contigs were longer than 3000 bp. Size distribution of the contigs is shown in S1 Fig. Finally, using the method of De Bruijn graphing and sequencing read information, we identified 107,259 transcripts with a mean length of 941.77 bp, transcripts ranged in length from ~200–3000 bp, identifying 49,615 transcripts with a length larger than 500 bp. We obtained 73,090 unigenes with a mean length of 693.24 bp. The lengths of 25,718 and 13,668 unigenes were larger than 500 bp and 1000 bp, respectively, while 64.82% of the unigenes had lengths between 0 to 500 bp (Fig 1). This result indicates that the length distribution of the transcripts and unigenes were represented in majority by short sequences with relatively little redundancy, which is similar to transcriptome analysis reported for other insect species using the same technology [10–14]. Importantly the longer sequences contribute only for 7.09% of the *M*. *alternatus* transcriptome, the majority of transcripts and unigenes were still less than 500 bp after assembly; this is probably due to the capacity of shorter sequences and low coverage of the transcriptome [5, 15]. A large number of assembled sequential data could provide a more deeply transcriptome information for future research, allowing rapid characterization for most of the transcripts and a reference for the genes of interest [15].

### Annotation of predicted proteins

**Fig 2:**
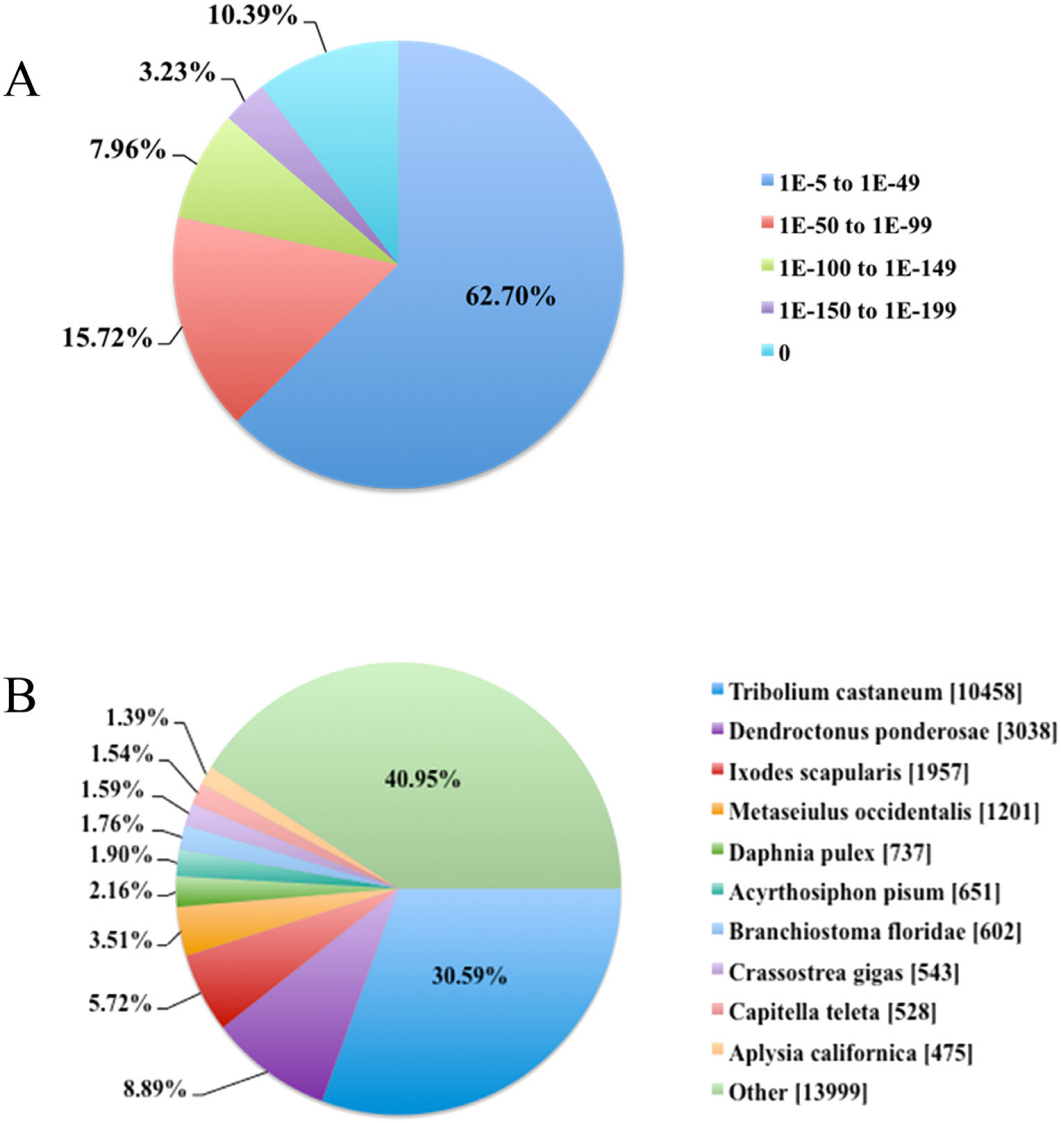
Characteristics of the homology search of Illumina sequences against the NR database. (A) E-value distribution of the BLAST hits for each unique sequence with a cut-off E-value of 1.0E^-^5. (B) Species distribution of the BLASTX results. The first hit of each sequence was used for further *in silico* analysis.

All assembled unigenes were used as an input for NR, Swiss-Prot, Gene Oontology (GO), Clusters of Orthologous Groups (COG), KOG and KEGG databases. BLAST and HMMER parameter E-values were set at 10^−5^ and 10^−10^ respectively, we were able to obtain annotated information for 36,828 unigenes, representing 50.38% of the unigenes. The rest of the unigene sequences (49.62%) had no significant matches in the existing databases. Unigenes comparison with the NR database produced 34,702 hits, the distribution of E-values demonstrated that 26.91% of the mapped sequences have strong homology (smaller than 1.0E^-49^) with an annotated sequence, and 62.70% of the homolog sequences ranged from 1.0E^-5^ to 1.0E^-49^ (Fig 2A). Based on the best species match, we found that *M*. *alternatus* sequences have 30.59% and 8.89% matches with sequences from the *Tribolium castaneum* and *Dendroctonus ponderosae*, both belonging to Coleopteran order, while only <6% matched to other insects (Fig 2B). Therefore, *M*. *alternatus* have the closest evolutionary distance with *T*. *castaneum*.

### GO assignments

**Fig 3:**
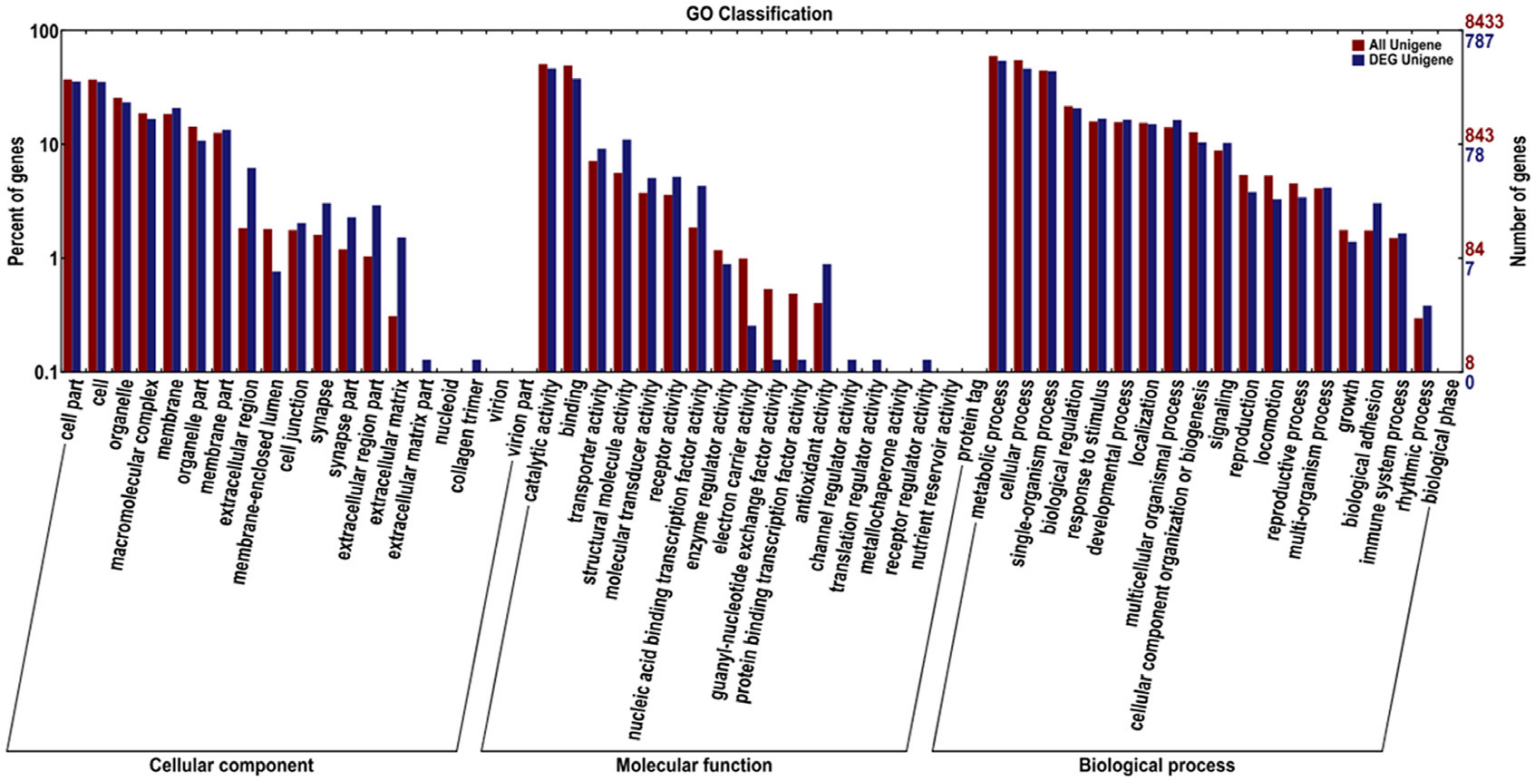
Distribution of second level GO of *Monochamus alternatus* Hope transcriptome. Distribution of GO categories assigned to the *Monochamus alternatus* Hope transcriptome. Unigenes were annotated in three categories: cellular components, molecular functions, and biological process. Right y-axis indicates the number of genes in a category; left y-axis indicates the genes percentage in a specific.

GO database is an internationally standardized gene function classification system, which provides a suitably updated standard vocabulary to describe the functional attributes of genes and gene products in an organism [16]. Transcript sequences were used as an input in the GO database; using BlastX a total of 105,612 unigenes were assigned to GO terms (Fig 3, S1 Table). According to the standard GO terms and 60 subcategories, differential gene expression and all unigenes from *M*. *alternatus* larva were statistically classified into three main GO categories: biological process, cellular component, and molecular function. Biological process represented the majority of GO annotations (52,883, 50.07%), followed by cellular component (29,970, 28.38%) and molecular function (22,759, 21.55%). The metabolic process (20.48%) and cellular process (18.55%) were predominant within the biological process category, indicating that the analyzed tissue has a high degree of metabolic activity; with the following subcategories: single-organism process (15.34%), biological regulation (8.05%), developmental process (5.59%), response to stimulus (5.49%), localization (5.38%), multicellular organismal process (5.12%), cellular component organization or biogenesis (4.26%) and signaling (3.16%). Biological processes contain most major cellular processes, from transportation and cell formation to transcription, translation and supersession. According to the classification of the cellular components, cell part (21.55%), cell (21.39%), and organelle (14.85%) are the most representative subcategories. Most of the annotated unigenes from the cellular component category, correspond to plastids and mitochondria. We also identified genes involved in the synthesis of secondary metabolites, and were grouped into catalytic activity (41.61%), binding (39.45%), transporter activity (5.63%), structural molecule activity (2.86%), molecular transducer activity (2.76%), receptor activity (2.43%) and nucleic acid binding transcription factor activity (2.02%), etc. A previous study reported similar classifications related to metabolic processes for *Tomicus yunnanensis* transcriptome [15]. GO annotations describe the contour features of the overall gene expression of *M*. *alternatus*, and revealed expressed genes encoding diverse structural, stress and regulatory proteins.

### COG classification

**Fig 4:**
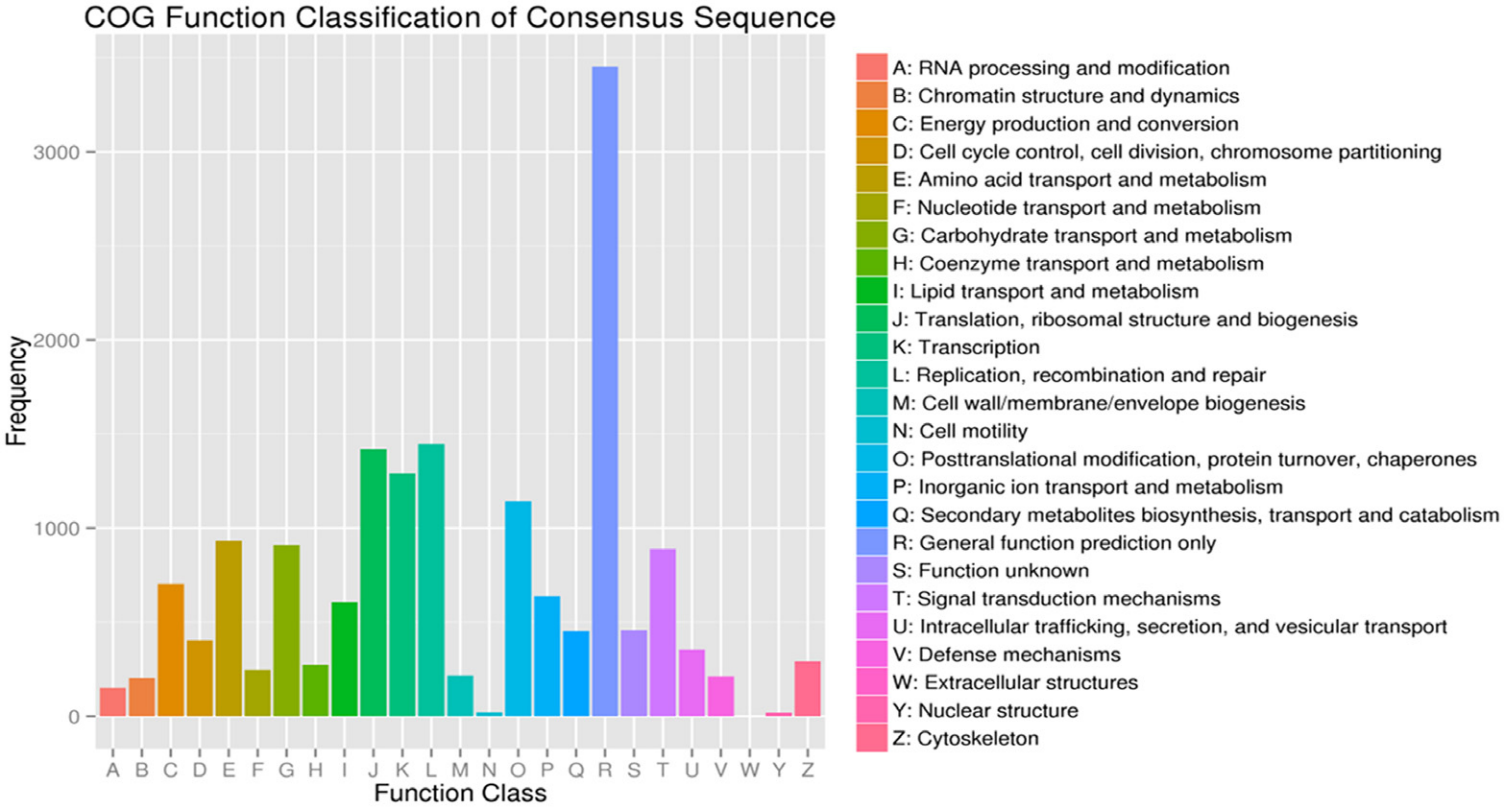
COG function classification of *Monochamus alternatus* Hope transcriptome. All putative proteins were analyzed using the COG database. COG classifications were divided into 25 functional categories; 16,730 classified unigenes were assigned to 25 COG classifications.

In total, information for 16,730 classified unigenes was obtained in the COG database (Fig 4). COG classifications were divided into 25 functional categories. Among these categories, general function prediction (19.90%) was the largest group, followed by translation, ribosomal structure and biogenesis (10.19%), posttranslational modification, protein turnover, chaperones (8.29%) and transcription (7.57%). Secondary metabolites biosynthesis, transport and catabolism represented 2.71%, given the relative importance of secondary metabolic activity for insect resistance. To some extent, COG classifications further reveal the potential specific reactions and the functional participation in molecular processes for genes expressed in *M*. *alternatus*.

### KEGG analysis

The KEGG Pathway database, is a collection of graphical maps representing different cellular processes, to systematically analyze metabolic pathways and functions of gene products in a cell [17]. To identify the represented biological pathways in *M*. *alternatus*, 34,302 annotated unigenes were analyzed using KEGG database. The results indicated that 13,024 unigenes matched with 224 KEGG pathways. These pathways are summarized in S2 Table. The top 10 pathways were Protein processing in endoplasmic reticulum (503 members), Ribosome (486 members), Spliceosome (444 members), RNA transport (415 members), Purine metabolism (410 members), Pyrimidine metabolism (321 members), Ribosome biogenesis in eukaryotes (312 members), Endocytosis (305 members), Oxidative phosphorylation (302 members) and Ubiquitin mediated proteolysis (288 members). These annotations provide powerful information for our research related specific biological processes and pathways of *M*. *alternatus*.

### Putative insecticide resistance-related genes

#### 1. Cytochrome P450 (P450)

Research over the past 10 years has provided clear evidence that in insects, P450 is involved in a number of physiological functions, such as hormone metabolism [18–20], adaptability of parasitic plants [21, 22] and resistance to insecticides [23, 24]. Approximately 222 types of P450-related unigenes were identified in *M*. *alternatus* transcriptome (S3 Table). We identified 103 P450-related sequences with a length bigger than 600 bp (46.40% of the global pests in the database); approximately half of the unigene sequences were long sequences (600 bp). In addition, the length of 30 P450-related sequences was larger than 1800 bp (13.51%) (Table 2). P450 genes identified from the *M*. *alternatus* transcriptome was comparable in number to those from *T*. *yunnanens*is transcriptome, although the final number of genes still needs to be verified via gene cloning [15]. Ai Junwen *et al*. classified the P450 enzyme system into four large families: CYP2, CYP3, CYP4 and mitochondrial P450, based on the phylogenetic analysis of *Drosophila* and silkworm P450 gene homology [25].

**Table 2:** Putative P450 genes identified in *Monochamus alternatus* Hope.

#Gene ID	Length	TFPKM	E_value	Identity (%)	Annotation
c40573.graph_c0	1814	1.16	0	69.31	PREDICTED: similar to cytochrome P450 CYP6BK17 [Tribolium castaneum]
c40753.graph_c0	1816.61	4.14	4.47E-179	56.8	cytochrome P450 [Leptinotarsa decemlineata]
c30543.graph_c0	1820	62.62	0	67.41	cytochrome P450 CYP4g56 [Dendroctonus ponderosae]
c31302.graph_c0	1838	6.33	4.52E-140	54.2	PREDICTED: similar to Cytochrome P450 315a1, mitochondrial precursor (CYPCCCXVA1) (Protein shadow) [Tribolium castaneum]
c42017.graph_c0	1863.46	94.8	0	58.7	cytochrome P450 [Leptinotarsa decemlineata]
c40340.graph_c0	1890	6.08	4.37E-146	53.67	cytochrome P450-like protein [Tribolium castaneum]
c41067.graph_c0	1893.65	52.99	7.25E-173	57.23	cytochrome P450 [Leptinotarsa decemlineata]
c41279.graph_c0	1927.2	5.72	2.73E-143	53.14	cytochrome P450-like protein [Tribolium castaneum]
c36280.graph_c0	1929	0.98	2.88E-177	58.92	hypothetical protein YQE_06277, partial [Dendroctonus ponderosae]
c31219.graph_c0	1933	450.22	0	86.12	cytochrome P450 [Leptinotarsa decemlineata]
c36851.graph_c0	1934.48	10.03	4.08E-153	49.5	PREDICTED: similar to antennae-rich cytochrome P450 [Tribolium castaneum]
c41193.graph_c0	1938.57	3.47	0	77.51	PREDICTED: similar to Cyp49a1 [Tribolium castaneum]
c33148.graph_c0	1948.26	7.19	1.00E-108	45.96	PREDICTED: cytochrome P450 4c3-like [Metaseiulus occidentalis]
c40989.graph_c0	1969	2.07	9.89E-157	50.39	cytochrome P450 6BQ7 [Tribolium castaneum]
c32887.graph_c0	1976	2.59	8.79E-121	40.76	cytochrome P450 monooxygenase [Panonychus citri]
c40370.graph_c0	1980.74	108.63	1.61E-170	53.98	cytochrome P450 9Z4 [Tribolium castaneum]
c37941.graph_c0	1993.46	28.81	3.61E-154	53.13	cytochrome P450 [Leptinotarsa decemlineata]
c39854.graph_c0	2006.71	796.85	0	64.19	cytochrome P450 9Z4 [Tribolium castaneum]
c30479.graph_c0	2050	14.46	1.07E-177	60.12	cytochrome P450 CYP314A1 [Tribolium castaneum]
c40839.graph_c0	2080	0.59	2.97E-159	53.91	cytochrome P450 6BQ7 [Tribolium castaneum]
c32624.graph_c0	2097.79	8.71	5.61E-93	54.24	hypothetical protein TcasGA2_TC006764 [Tribolium castaneum]
c41739.graph_c1	2128.53	108.31	6.44E-173	53.73	cytochrome P450 [Leptinotarsa decemlineata]
c37224.graph_c0	2131	15.25	1.13E-153	51.11	PREDICTED: similar to antennae-rich cytochrome P450 [Tribolium castaneum]
c40116.graph_c0	2135	0.35	2.85E-173	56.86	cytochrome P450 [Leptinotarsa decemlineata]
c38458.graph_c0	2434	36.65	3.33E-173	55.34	cytochrome P450 [Leptinotarsa decemlineata]
c29697.graph_c0	2490.42	5.88	0	64.56	cytochrome P450, putative [Ixodes scapularis]
c42120.graph_c0	2513.73	25.61	2.28E-147	50.1	cytochrome P450 CYP6CR2 [Dendroctonus ponderosae]
c40510.graph_c0	2515.24	10.43	4.67E-100	41.25	PREDICTED: similar to cytochrome P450 monooxygenase [Tribolium castaneum]
c39155.graph_c0	2599.72	266.53	0	84.85	PREDICTED: similar to nadph cytochrome P450 [Tribolium castaneum]
c40802.graph_c0	2692.71	0.6	7.00E-146	56.42	cytochrome P450 353A1 [Tribolium castaneum]

Previous studies have reported that 17 P450 genes from CYP3 and CYP4 families are associated with plant toxins and pesticide resistance [25,26]. Based in phylogenic analyses; we found evidence that CYP1, CYP3, CYP4 and mitochondrial families are present in *M*. *alternatus* transcriptome. We also identified CYP1A1, which belongs to the CYP1 family, and CYP3A4, CYP3A5 and CYP3A7, which belong to the CYP3 family. Interestingly, the P450 genes identified in *M*. *alternatus* differ from those reported in *T*. *castaneum* and other insect systems.

Recent studies have shown that P450 has an increased expression in insecticide resistant insects; moreover, there is also evidence for P450 gene duplication and amplification in four types of insects *in vivo* [27]. However, there is no experimental data to support the importance and biological role of P450 complex related to insecticide resistance in *M*. *alternatus*.

#### 2. Glutathione S-transferase (GST)

GSTs specifically catalyze glutathione thiol and interact with other electrophilic groups [28, 29]; GST is one of the main detoxification enzymes in insecticide metabolism. GST high levels of expression are related to insecticide resistance mechanisms in insects [27]. We identified 96 GST unigenes in *M*. *alternatus* transcriptome. The length of 19 GST-related sequences was greater than 1000 bp (19.79%) (Table 3). GSTs are divided into three major categories according to their cellular location: cytosolic, microsomal, and mitochondrial [30]. Cytosolic matrix GSTs in insects are further divided into at least six classes (delta, epsilon, omega, sigma, theta, and zeta) based on sequence homology of the N-terminus, substrate specificity, immunoreactivity, and sensitivity to different inhibitors [31–33]. Delta and epsilon classes are unique in insects [34]. We found delta, omega, and theta types of GST in *M*. *alternatus* transcriptome, however we could not identify any gene from the epsilon class. Previous studies have reported the identification of sigma, delta and theta GST classes in Nasonia vitripennis; epsilon, sigma, omega, and delta in *T*. *castaneum*; and one delta unigene in *T*. *yunnanensis* [35, 36]. The GST genes identified in *M*. *alternatus* transcriptome can contribute to a greater understanding of the relationship between GSTs and insecticide resistance in insects.

**Table 3:** Putative identified GST genes in *Monochamus alternatus* Hope.

#Gene ID	Length(bp)	FPKM	E_value	Identity(%)	Annotation
c10463.graph_c0	1125.51	3	2.16E-06	30.98	unnamed protein product, partial [Leishmania mexicana MHOM/GT/2001/U1103]
c8990.graph_c0	1175.51	2.25	6.02E-15	31.75	predicted protein [Nematostella vectensis]
c44708.graph_c0	1277.51	1.85	1.99E-67	44.01	elongation factor 1-gamma [Cryptosporidium hominis TU502]
c36023.graph_c0	1307.03	4.98	1.15E-47	44.93	glutathione transferase delta-like Yv4019D08 [Sarcoptes scabiei type hominis]
c27703.graph_c0	1919.09	18.56	3.85E-106	49.22	failed axon connections, putative [Ixodes scapularis]
c21401.graph_c0	1080.51	6.46	7.11E-84	51.77	PREDICTED: similar to metaxin 1 [Tribolium castaneum]
c29135.graph_c0	3037.97	2.24	2.39E-102	54.39	PREDICTED: lachesin-like [Metaseiulus occidentalis]
c41606.graph_c0	2305.51	4.56	0	60.52	glutathione S-transferase C-terminal domain-containing protein [Tribolium castaneum]
c39852.graph_c0	3260.51	8.74	0	62.51	PREDICTED: similar to AAEL014709-PA [Tribolium castaneum]
c41863.graph_c0	1835.51	0.67	6.55E-150	66.07	hypothetical protein YQE_09889, partial [Dendroctonus ponderosae]
c33923.graph_c0	1133.51	109.27	9.20E-98	67.9	unknown [Dendroctonus ponderosae]
c35423.graph_c0	1148.51	48.56	3.94E-96	68.46	unknown [Dendroctonus ponderosae]
c30871.graph_c0	1594.51	10.77	1.21E-88	69.49	glutathione transferase mu class Yv5001D03 [Sarcoptes scabiei type hominis]
c10168.graph_c0	1176.51	55.03	7.72E-87	71.5	glutathione-S-transferase theta, GST, putative [Pediculus humanus corporis]
c39366.graph_c0	1552.51	10.74	3.40E-120	79.54	PREDICTED: similar to metaxin 2 [Tribolium castaneum]
c10006.graph_c1	1441.51	2940.6	0	82.28	PREDICTED: similar to Elongation factor 1-gamma (EF-1-gamma) (eEF-1B gamma) [Tribolium castaneum]
c35156.graph_c0	2321.46	37.05	2.41E-102	82.95	PREDICTED: similar to AGAP008106-PA [Tribolium castaneum]
c33364.graph_c0	2144.51	273.41	0	84.07	PREDICTED: similar to failed axon connections protein [Tribolium castaneum]
c40777.graph_c0	1429.64	32.41	0	86.15	PREDICTED: similar to SVOP protein [Tribolium castaneum]

### Insecticide receptors and resistance-related genes

**Fig 5:**
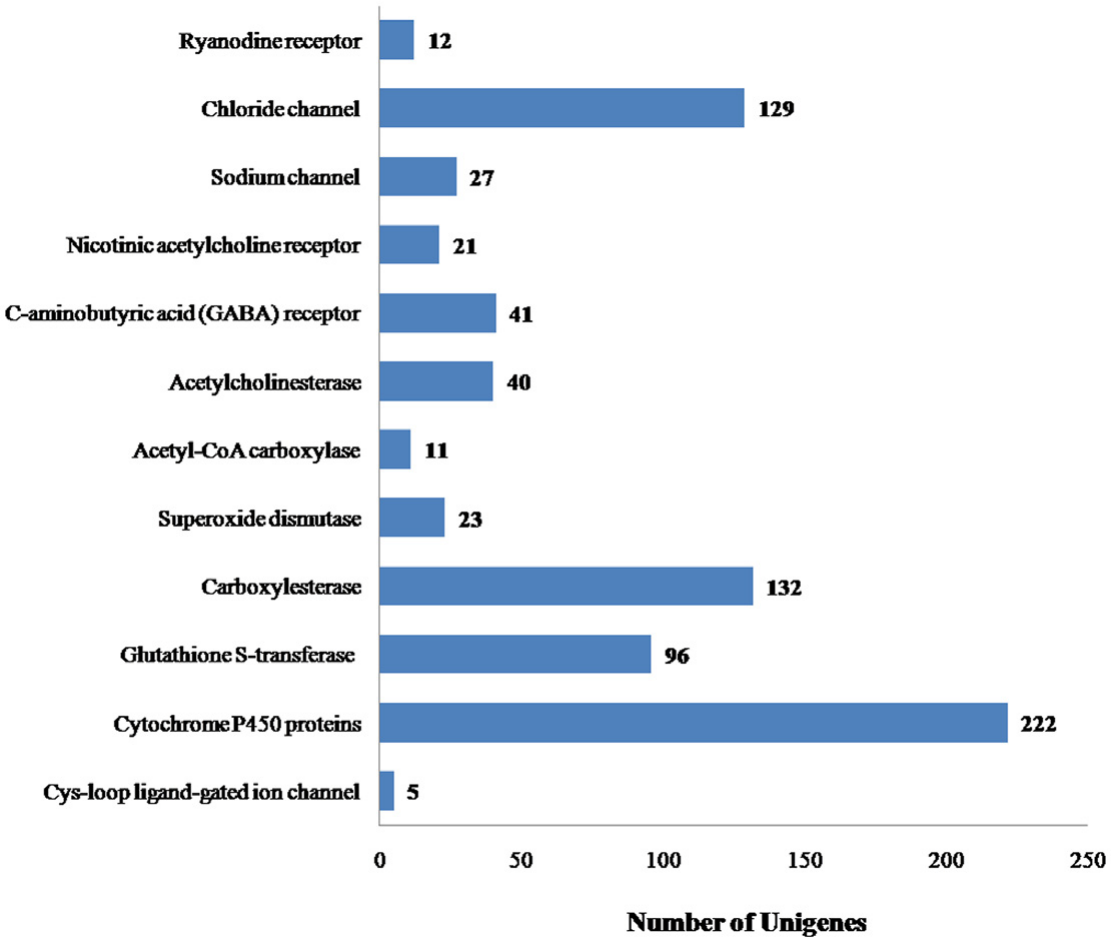
Number of unigenes related to pesticide receptors and resistance-related genes. x-axis indicates the number of unigenes, y-axis indicates the specific unigenes related to pesticide receptors and resistance-related genes. Seven hundred and fifty-nine insecticide receptors and resistance-related unigenes were identified in *M*. *alternatus* transcriptome.

In addition to the identified genes described above, we identified 384 unigenes that represent potential pesticide receptors and insecticide resistance-related genes; including FigCys-loop ligand-gated ion channel (Cys-loop LGIC), carboxylesterase, superoxide dismutase, acetyl-CoA carboxylase, acetylcholinesterase, c-aminobutyric, acid (GABA) receptors, nicotinic acetylcholine receptors, sodium channels, chloride channels and ryanodine receptors (Fig 5). Further study of these genes could uncover potential insecticide receptors and provide the bases to test whether these genes play functional roles in insecticide resistance.

### RNA interference-genes

RNA interference (RNAi) is an important pathway that is used in many organisims to regulate gene expression [37]. RNAi pathways have been indentified in *Drosophila melanogaster* [38–40], *T*. *castaneum* [41–46] and *Bombyx mori* [47–49].

**Fig 6:**
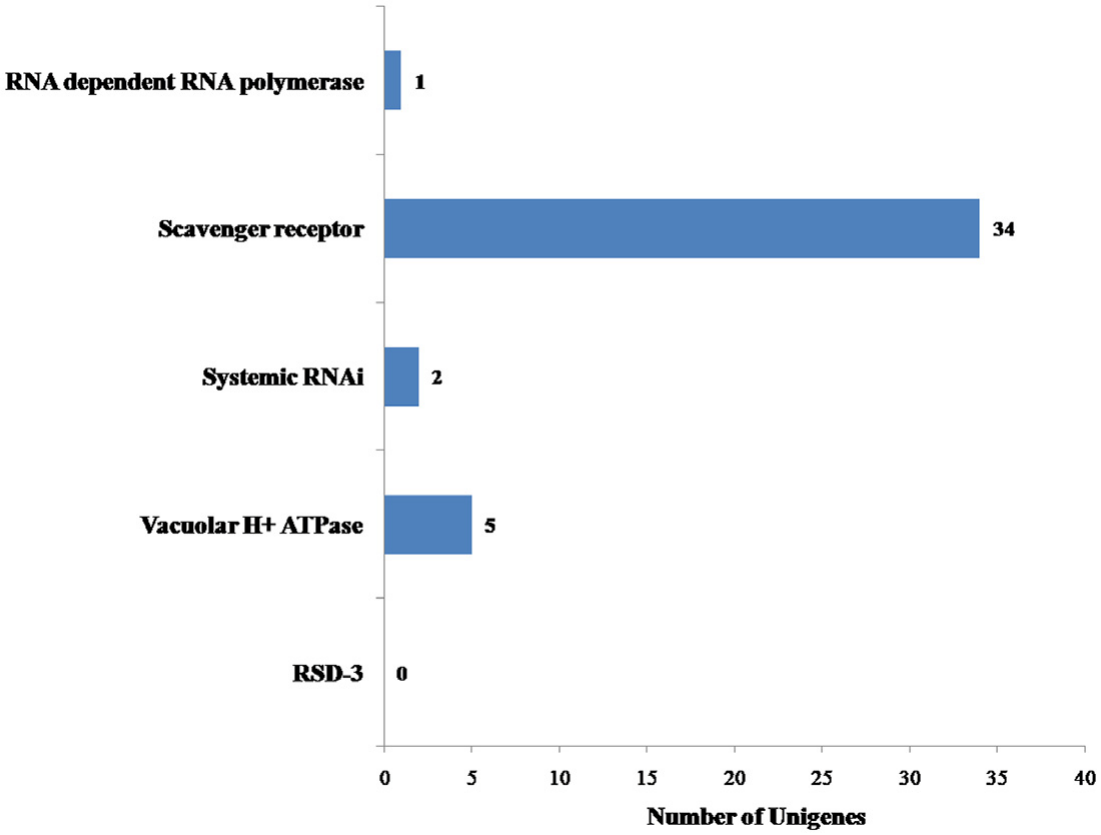
Number of unigenes related to RNAi. x-axis indicates the corresponding number of unigenes and y-axis indicates the specific unigenes related to RNAi. Forty-two unigenes coding for RNAi identified in the database, 26 of them were larger than 600 bp (61.90%) and 23 were more than 1 kb (54.76%).

Forty-two unigenes related to RNAi pathways were identified (Fig 6); we identified two SID-1, 34 scavenger receptors, Figone RNA-dependent RNA polymerase, and five vacuolar H^+^ ATPase unigenes in *M*. *alternatus* transcriptome. These components have previously been reported as part of the RNAi uptake mechanisms in insects [50]. Interestingly, we did not identify any RSD-3 unigenes in the transcriptome, although its participation in RNAi pathways. Among the identified genes, 26 unigenes were larger than 600 bp (61.90%) and 23 were more than 1 kb (54.76%). Moreover, two SID-1-related unigenes were both represented by longer sequences in the transcriptome, at c38753.graph_c0 (2731.42 bp) and c41338.graph_c0 (3066.51 bp), respectively.

SID-1 is a multispan transmembrane protein that is crucial for systemic RNAi pathways in *Caenorhabditis elegans*, it delivers dsRNAs to cells [50]. However, SID-1 has not been described in *D*. *melanogaster*, indicating that the existence or absence of *sid-1* gene could play an important part in determining whether systemic RNAi is present in the biome [51]. Although a sid-1 ortholog has also been found in the cotton aphid *Aphis gossypii* [52], further research is still needed to provide evidence for the molecular basis of systemic RNAi in *M*. *alternatus*.

Scavenger receptors can recognize extensive polyanionic ligands, and they play key roles mediating phagocytosis of pathogens in *Drosophila* [50]. Among the 34 identified unigenes related to scavenger receptors, nine of them had lengths above 1 kb, while the rest ranged from 100 bp to 1 kb. However, we were not able to identify important relevant sequences homologous sequences to the scavenger receptors of mammals [53, 54], but we were able to identify them by homology to the flesh fly and C. elegans.

Finally, we identified five vacuolar H^+^ ATPase unigenes and one RNA-dependent RNA polymerase-1 unigene. Previous studies have found that vacuolar H+ ATPase-deficient S2 *Drosophila* cells accumulate dsRNA in endocytic vesicles and showing an endocytosis-based mechanism as a way to disrupt vacuolar H^+^ ATPase in the S2 cells to induce RNAi silencing [55].

The study of SID-1, scavenger receptors, vacuolar H^+^ ATPase, RSD-3 and RNA-dependent RNA polymerase genes can deepen our understanding of the biology of defense against parasitic endogenous nucleic acids and exogenous pathogen nucleic acids and provides a basis for the expression of regulatory protein coding genes [56, 57].

### Potential *Bacillus thuringensis* (Bt) receptors

**Fig 7:**
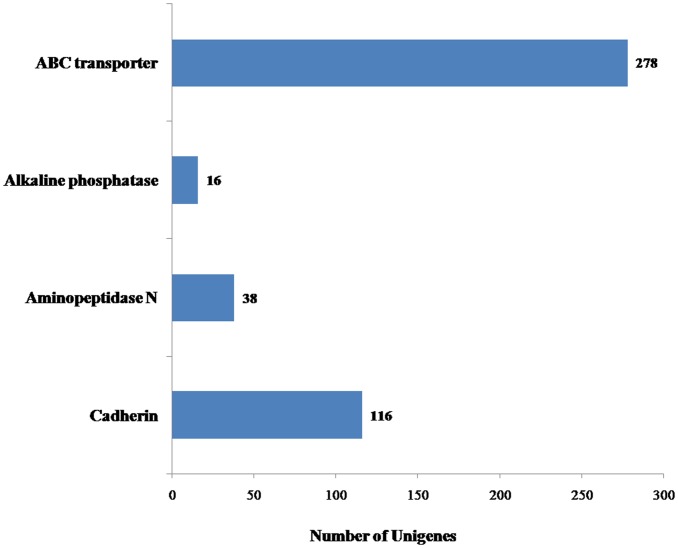
Number of unigenes related to Bt receptors. x-axis indicates the corresponding number of unigenes and y-axis indicates the specific unigenes related to Bt receptors. Four hundred and forty-eight Bt receptor unigenes were confirmed in the *Monochamus alternatus* Hope transcriptome through *in silico* comparisons.

Receptor molecules related to B. thuringensis Cry toxin mechanisms, have been characterized in the the insect midgut; they have been widely studied, most notably: aminopeptidase (APN), alkaline phosphatase (ALP) and cadherin [58, 59]. We identified a total of 448 Bt receptor unigenes in *M*. *alternatus* transcriptome, including: ALP, APN, cadherin and the ABC transporter, which are currently the four described Bt insect receptor molecules (Fig 7).

APN [60–62] and cadherin [63] are considered the most important type of receptors within the putative insect Cry receptors. *M*. *alternatus* transcriptome revealed 38 APN unigenes and 116 cadherin unigenes, representing both approximately 8% of the total number of related Bti-receptor unigenes. APNs belong to the zinc-binding metalloprotease/peptidase enzymes and are anchored to the midgut membrane through GPI anchors [64]. Crava *et al*. clustered APNs into seven classes based on phylogenetic analyses of lepidopteran, Hughes found that in intestinal tissue, APN1 class was the most highly expressed within a complex mix of APN expression data [65, 66]. Cadherins are mainly localized in adherens junctions and are involved in cell adhesion [59]; they have been found anchoring midgut epithelial cells of *Manduca sexta* and *Lymantria dispar* larvae. We found that the number of identified cadherin unigenes contributed in nearly 26% of the total number of Bt receptor molecules in *M*. *alternatus*, second only to ABC transporters (278, ~62%). It has previously been reported that APN and cadherin-like (CAD-like) midgut proteins in lepidotera can interact with Cry1 toxins. In Diptera, homologous APN, CAD-like, and alkaline phosphatase proteins of mosquitoes are also considered as Cry11 and Cry4 receptor proteins. In the longicorn of coleopterous, a cadherin-like protein acts as Cry3Aa receptor; finally, gene silencing has confirmed that APN and CAD-like proteins are the most representative Cry receptors [67]. In insects, ALPs are a major group of Cry-binding proteins; their roles as receptor molecules have extensively studied in Lepidoptera, Coleoptera and Diptera larvae [68–71]. Data suggest that the interaction between Cry1Ac and ALP affects the midgut protease activity during the incubation period in *Heliothis virescens* and *M*. *sexta* larvae [62, 68]. *Monochanus alternatus* transcriptome revealed 16 ALP unigenes, which contribute to only ~3% of the Bt receptor-related unigenes. Using RNAi silencing of APN and ALP genes in *M*. *sexta* larvae, Flores-Escobar *et al*. found that for Cry1Ab toxicity, binding to ALP was more important than APN, however for Cry1Ac, APN was more important, these results suggest that Cry binding receptors have specific affinity for different Cry toxins [72].

In addition to ANP, ALP and cadherins, *M*. *alternatus* transcriptome revealed 278 ABC transporter-related unigenes, contributing for 62.05% of the total number of Bt receptor-related unigenes. The ABC family in insects is related to multi-drug resistance [73]. Recently, ATP-binding cassette transporter subfamily C member 2 (ABCC2) was identified as a Cry1 toxin receptor in *B*. *mori* larvae [74], with a consistent role in the mechanism of Cry1 toxin resistance [75, 76].

### Intestinal digestive enzymes

Midgut proteases play an important role in activating Cry toxins, producing the toxin’s core 3D structure in the insect midgut [77] and the type and abundance of insect proteases are important for toxin specificity [78]. Changes in the content and activity of proteases can lead to resistance [78]. Frederick S. Walters *et al*. found that mCry3A toxicity to corn rootworm larvae was attributed to a chymotrypsin/cathepsin G site, which enhances cleavage and subsequent binding of the activated toxin to midgut cells [79].

**Fig 8:**
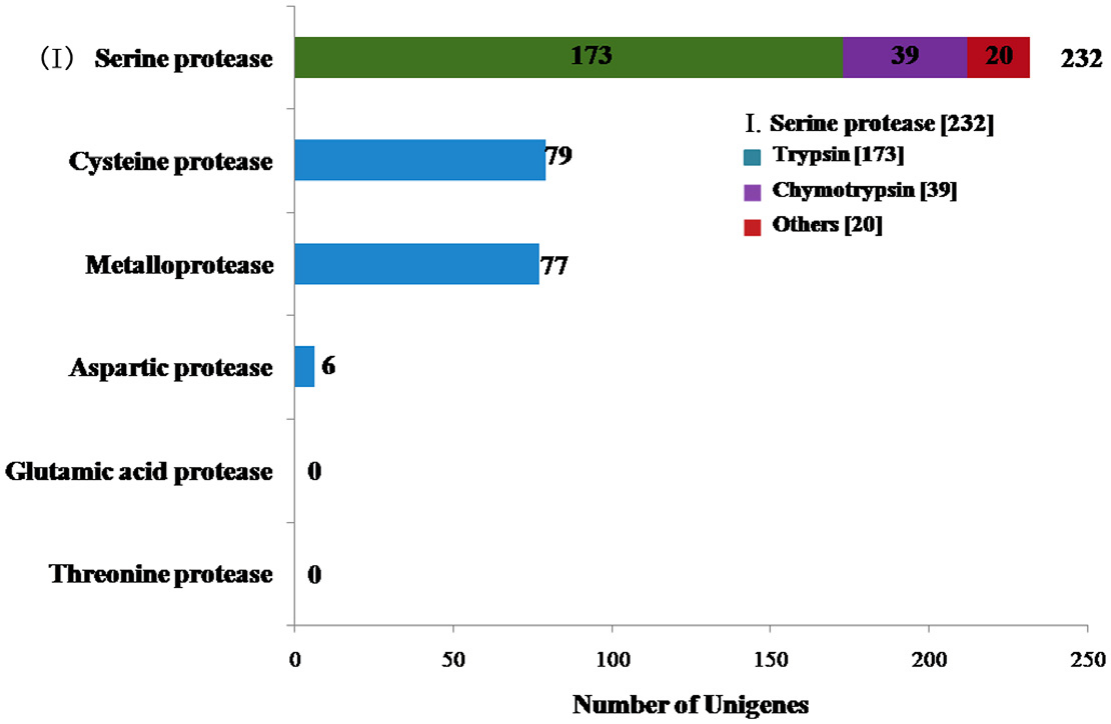
Number of unigenes related to intestinal digestive enzymes. x-axis indicates the corresponding number of unigenes and y-axis indicates the specific unigenes related to intestinal digestive enzymes. Three hundred and ninety-four protease-related unigenes were identified in *M*. *alternatus* Hope transcriptome. **(I) Number of specific unigenes related to serine proteases in intestinal digestive enzymes**. The icon indicates specific unigenes. The number in brackets indicates the corresponding number of unigenes.

In Lepidoptera and Diptera, serine proteases are the main type of intestinal protease [80, 81]; meanwhile in Coleoptera, cysteine and aspartic proteases are the principal class of digestive enzymes. Cathepsin G serine protease has been considered as the principal enzyme because its activity is a key step towards Cry toxicity. It has been demonstrated that cathepsin activity can be Cry toxin specific, for example, *Acyrthosiphon pisum* mainly expresses cathepsin L and cathepsin B types [82], but different serine proteases have activity for Cry4A and Cry4B in sensitive Diptera. [80]. Moreover, cathepsins B, L and serine peptidases such as trypsins and chymotrypsins are the major enzyme components in the larval midgut of tenebrionid beetles [83]. In general, the digestive protease activity of Coleopteran insects depends mainly on cysteine proteases [84, 85]. We found 394 protease-related unigenes in *M*. *alternatus* transcriptome (Fig 8). Among these unigenes, serine proteases were the most represented group (232, 58.88%), followed by cysteine proteases (79, 20.05%), metalloproteases (77, 19.54%) and aspartic acid proteases (61.52%). Threonine proteases and glutamic acid proteases were not found in our data. Interestingly, we found 212 serine proteases genes (Fig 8). Only 82 serine protease genes had lengths above 1 kb. The unigenes with lengths less than 1 kb were represented by 95 trypsin and 28 chymotrypsin genes. Serine proteases are the main protein digestive enzymes, accounting for 95% of the digestive activity in Lepidoptera [86]. In insects, genes encoding serine proteases (SP) and serine protease homologs (SPH) comprise a large family of proteins involved in digestion, immune defense, development and other process [87]. Cysteine proteases play an important role as virulence factors in *Entamoeba histolytica*. Furthermore, from invasive trophozoites to parasites in dormant infective cyst stages, cysteine proteases have an important role in parasite morphology [88]. It has been reported that Colorado potato beetle (CPB) midgut membrane metalloproteases participate in the proteolytic processing of Cry3Aa toxin [89]. C. Rausell *et al*. found that Brush border membrane vesicles (BBMV)-associated metalloproteases can cut Cry3Aa toxin specificity, thus significantly reducing the activity of pore formation [90]. In vertebrates, the four main aspartic proteases are pepsins, cathepsin D, cathepsin E and renins [91–93], these enzymes mainly degrade endogenous proteins. Our data confirmed the abundance of protease-related unigenes in *M*. *alternatus* transcriptome, which has significant implications for future research regarding the mechanism of action of Cry toxin in *M*. *alternatus*. The information provided by insect transcriptomes can contribute in understanding of digestive enzyme functions, in addition to generate new tools to improve the activity of proteases.

### Possible future insect control targets

**Fig 9:**
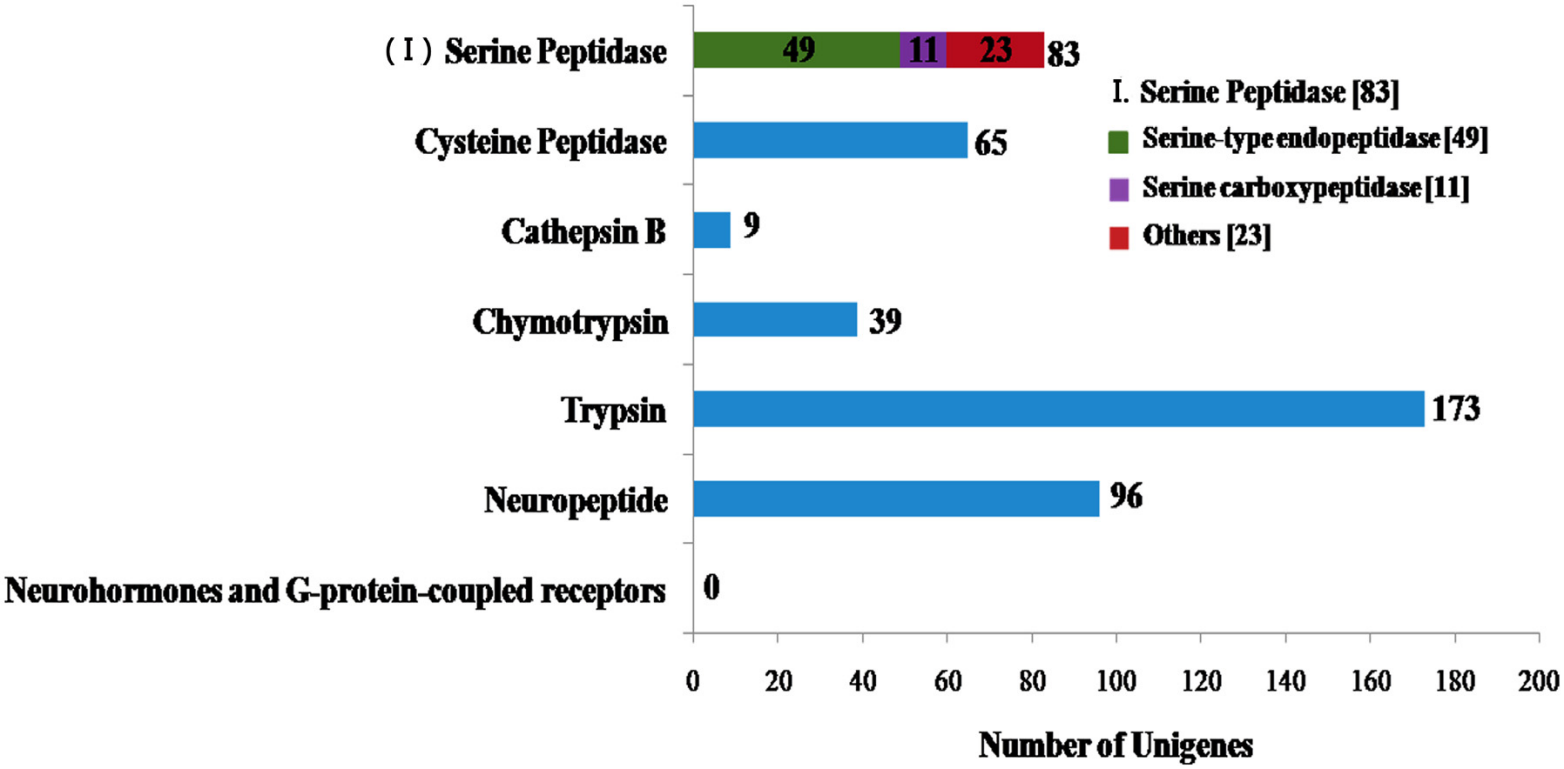
Number of unigenes related to possible future insect control targets. x-axis indicates the corresponding number of unigenes and y-axis indicates the specific unigenes related to possible future insect control targets. 465 possible future insect control targets unigenes were found in *M*. *alternatus* Hope transcriptome. Among these, 117 unigenes had a length above 600 bp and 87 above 1 kb. **(I) Number of specific unigenes related to Serine Peptidase in possible future insect control targets**. The icon indicates specific unigenes. The number in brackets indicates the corresponding number of unigenes. Serine carboxypeptidase, serine-type endopeptidase and others belongs to the serine peptidases.

In addition to the diverse type of genes described above (Fig 9), we also identified candidate unigenes as targets for future insect control strategies, such as cathepsin B, cysteine peptidases, neuropeptides and serine peptidases. Among them, we found serine carboxypeptidase and serine-type endopeptidase, belonging to the serine peptidases (Fig 9). Of these, 117 unigenes had a length above 600 bp, 87 of which were above 1 kb. Moreover, we found 96 neuropeptide unigenes, corresponding to insect neurohormones that signal via G-protein-coupled receptors (GPCRs) to control growth, reproduction, behavior, breeding and other physiological processes [94]. Identifying important molecule-related unigenes for these basic physiological processes in *M*. *alternatus* will provide a reference for possible future research into targets for insect control and other applications.

### Immune-related molecules

**Fig 10:**
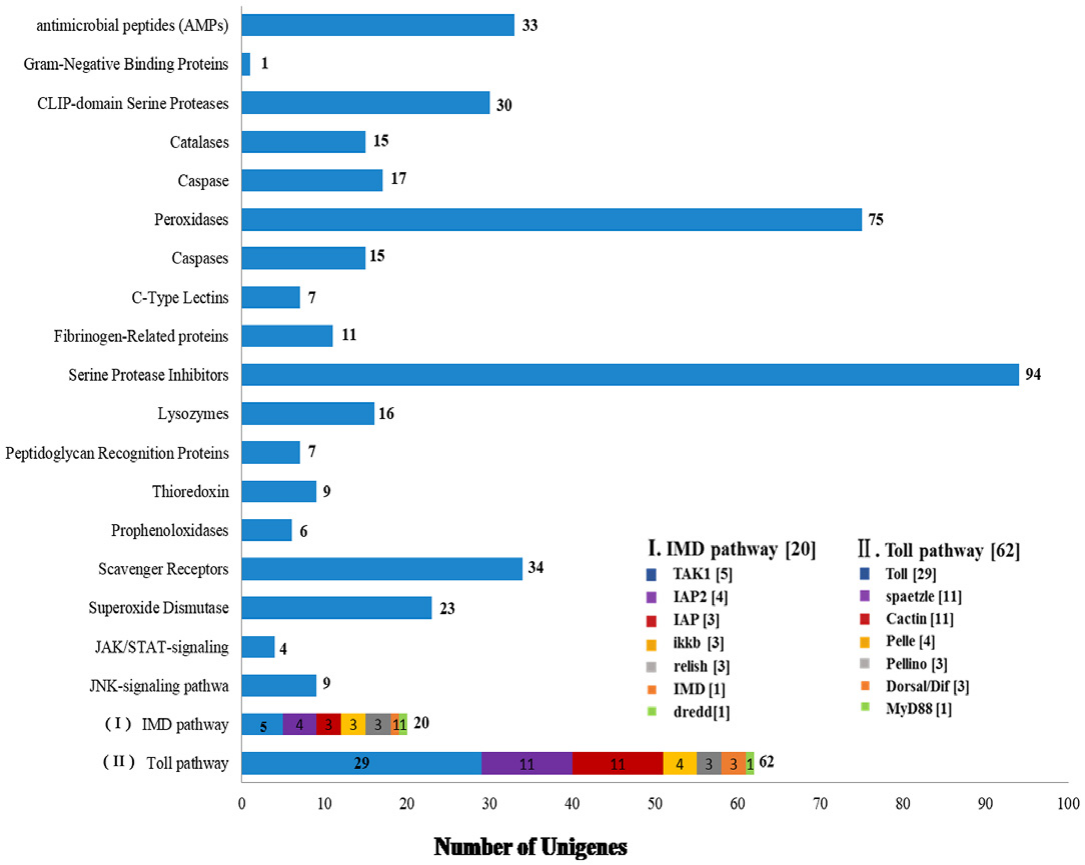
Number of unigenes related to immune-related molecules. x-axis indicates the corresponding number of unigenes and y-axis indicates the specific unigenes related to immune-related molecules. 478 unigenes were identified related to immune molecules and receptors in the transcriptome. This group contains 20 widely recognized immune factors. **(I) Number of specific unigenes related to IMD pathway in immune-related molecules. (II) Number of specific unigenes related to Toll pathway in immune-related molecules**. The icon indicates specific unigenes. The number in brackets indicates the corresponding number of unigenes.

Organisms have unique immune mechanisms against pathogens in the environment. Compared to vertebrates, invertebrates lack of an acquired immune system and rely on an innate immune system; adaptive immunity in insects has yet to be identified [95]. The innate immune system of insects is divided into cellular immunity and humoral immunity. Cellular immunity comprises phagocytosis, melanization and encapsulation. Humoral immunity has three steps:1) pattern recognition protein receptors (PRRs) recognize and bind to pathogen-associated molecular patterns (PAMPs); 2) a series of innate immune responses are activated, and 3) finally triggering the generation of innate effector activities (phagocytosis) and effector molecules (antimicrobial peptides (AMPs)) [96, 97]. RNAi machinery also plays an important role in regulating the innate immune response in insects and other organisms [98]. We identified 478 unigenes related to immune molecules and receptors related to immune activities (Fig 10). This group contains 20 widely recognized immune factors, including serine protease inhibitors (94, ~20%) and being the most abundant, followed by peroxidases (75, 16%). Interestingly, MD2(an extracellular binding partner of Toll-like receptor 4 (TLR4),)-like proteins and ML (MD-2-related lipid-recognition) -related unigenes were not found, although MD2-like gene family encodes for secretory proteins [99]. Therefore, molecules directly related to MLs may not be present in *M*. *alternatus* immune system.

#### 1. Antimicrobial peptides

AMP-related unigenes were represented by approximately 7% of the total identified immune factors. AMPs play important roles in the innate immune system of invertebrates and possess significantly broad antimicrobial activity. AMPs are widespread in nature and mainly distributed in Lepidoptera, Coleoptera, Diptera, Hymenoptera, Hemiptera, Isoptera, Homoptera and Odonata orders. Antimicrobial peptides such as metchnikowin, drosocin, defensin, diptericins, attacins, cecropins, drosomycins, have been characterized in *D*. *melanogaster* [100, 101]. We identified 33 antimicrobial peptide-related coding unigenes: antifungal peptides were the most abundant (15, ~ 45%), while attacins, cecropins and defensins represented 15%, 9% and 6%, respectively. Iijima R *et al*. isolated an anti-fungal peptide (AFP) from *Sarcophaga peregrina* as the first reported AFP [102], it is composed of 67 amino acid residues and is rich in glycine and histidine. We identified 5 attacin-related unigenes. Attacins only participate in bacteriostasis for some gram-negative bacteria [103]. At present, more than 20 cecropin analogues have been isolated from Lepidoptera and Diptera insects [104]. Cecropines form a voltage-dependent channel that changes the permeability of the bacterial cell membrane. Its antibacterial spectrum includes gram-negative and gram-positive bacteria. Moreover, we found two defensin unigenes (~6%), insect defensins were first isolated in *Phormia terranovae*, due to their high homology with mammalian defensins and later identified in *S*. *peregrina* (sapecins) [105]. Members of insect defensins family were also identified in *A*. *aegypti* and *A*. *gambiae* [106]. Although fewer in number defensin activities cannot be ignored in *M*. *alternatus* immune system. Dobson *et al*. hypothesized that the highly diverse function of antibacterial peptides have implications in the evolution of pathogen inhibition mechanisms in the host [107].

#### 2. Immune enzymes and inhibitors

We found 17 unigenes related to caspases (CASPs), a class of aspartic acid proteolytic enzymes containing cysteine and responsible for apoptosis initiation; they play a critical role in regulating cell death in the growth process of an organism. CASPs 4, 5 and 11 have been reported as cytoplasmic receptors for gram-negative lipopolysaccharides (LPS) [30, 108]. LPS are important structural components of gram-negative bacterial outer cell membranes and can activate the innate immune response through TLR4.

Ninety-four serine protease inhibitors (SRPN) unigenes were identified in our data, contributing for ~20% of the total number of immune factor unigenes; which may possess important active defense immune functions in the process of pathogenic microorganism infection. SRPNs can participate in a variety of physiological reactions and are present higher eukaryotes and viruses [109, 110].

#### 3. Oxidative stress molecules

In insect immune responses, a high level of reactive oxygen species (ROS) is produced. Peroxidase-related unigenes identified in *M*. *alternatus* contributed for 16% of the total number of immune factor-related unigenes, we identified 75 peroxidase-related unigenes, 21 unigenes corresponding to including glutathione [GPXs], 8 corresponding to thioredoxin [TPXs], and 30 unigenes corresponding to Haem. Furthermore, we identified 15 catalase-related unigenes (CAT), 16 Lysozyme-related unigenes (LYS), and 6 prophenoloxidases-related unigenes (PPO). CATs can effectively convert H_2_O_2_ into water and oxygen, removing hydrogen peroxide from the cell to prevent H_2_O_2_ poisoning. Therefore, CATs are key enzymes in the biological defense system. Studies have shown that CAT gene disruption in *D*. *melanogaster* quickly leads to death [111]. Moreover, we also identifed superoxide dismutases (SODs) unigenes (5%). SODs can convert •O_2_^--^ into hydrogen peroxide (H_2_O_2_), which is less toxic.

#### 4. Lectins and galectins

Seven unigenes related to C-Type lectins (CTLs) and five galectin (GALEs) genes were identified in our data. CTLs are secreted proteins or membrane proteins that depend on calcium ions for their function, they are the largest and most diverse family of animal lectins. Several invertebrate immune responses involving CTLs include opsonization [112, 113], pathogen elimination, hemocytes biosynthesis and activating prophendoxidase to produce melaninization [114–116]. GALEs are β-galactoside binding proteins that depend on mercaptans. There is evidence that galectins are related to congenital immunity in *Drosophila* and *An*. *gambiae* [117].

#### 5. Pattern recognition proteins

Peptidoglycan recognition proteins (PGRP) are pattern recognition receptors that can recognize bacteria peptidoglycans and they play an important role in recognition and adaption of innate immunity. We found 7 PGRP-related unigenes and interestingly, 34 pattern recognition scavenger receptor (SCR), SCR mediates the recognition of pathogen LPS and LTA [118–120] and “clean up” after apoptosis [53, 54, 121–125]. We hypothesize that SCR-associated molecules in *M*. *alternatus* may have similar actions. In addition, we identified 11 fibrinogen-related proteins (FREP) and 30 CLIP-domain serine proteases, contributing for 2% and 6% respectively. CLIP proteases have regulatory roles for immune responses in insects [111, 126–132]. We also discovered one unigene that correlated with a Gram-Negative Binding Protein (GNBP). Based on the genetics and biochemistry analysis GNBP1 has been identified as a co-receptor for gram positive microbes and PGRP-SA [133, 134]. Finally, we identified 9 thioester-containing (TEP) protein-related unigenes (~2%). The TEP family is mainly composed of vertebrate complementary factors (C3, C4, C5) and α2-macroglobulin. *Anopheles gambiae* TEP1 is a typical TEP in insects, which can bind to the surface of pathogenic bacteria and promote the haemocytes of mosquitoes to phagocyte the bacteria or eliminate *Plasmodium* [135–137].

#### 6. IMD, Toll, JAK/STAT and JNK-signaling pathway

We identifed unigenes that are closely related to the IMD, Toll, JAK/STAT and JNK-signaling pathway, represented by 20, 62, 4 and 9 unigenes, respectively.

#### Imd pathway

The Imd pathway is the primary immune pathway that acts against Gram-negative bacteria. We identified 20 unigenes, such as IMD, TAK1, IAP, IAP2, dredd, ikkb and relish (Fig 10). Imd pathway regulates the activity of a third *Drosophila* NF-kB protein named Relish, controls the expression of most of the *Drosophila* AMPs and, thus is indispensable for normal immunity [138]. TAK1 is ubiquitin-dependent kinase of IKK [68,139]. IAP2 participates in Relish nuclear localization in *Drosophila* [140]. IKK enhanced the phosphorylation of Relish, after Relish cleavage by DREDD, causing translocation of the N-terminal end to the nucleus [141].

#### Toll pathway

ixty-two identified unigenes were related to the Toll pathway, including spatzle, Toll, MyD88, Pelle, pellino, Cactin and Dorsal/Dif (Fig 10). In Drosophila the Toll receptors are essential for embryonic development and immunity. The induction of the Toll pathway by Gram-positive bacteria or fungi leads to the activation od cellular immunity and the systemic production of AMPs. The Toll receptor is activated when the proteolytically cleaved ligand Spatzle binds the receptor leading to the activation of the NF-kB factors Dorsal-related immunity factor [141, 142]. Cactus is phosphorylated by a complex consisting of MyD88, tube and pelle, causing degradation of cactus and release of Dorsal/Dif [143, 144]. Besides, pellino and Cactin genes showed high expression levels in *T*. *castaneum* transcriptome [145].

#### JAK/STAT-signaling pathway/ JNK-signaling pathway

two additional signaling pathways have been shown to have immune functions in insects: the JNK and JAK-STAT pathways involved in cell stress or wound response as other immune pathways in insects [139]. Only two genes involved in JAK-STAT signaling pathway were identified in our transcriptome sequencing data, including 3 unigenes related to STAT92E and 1 unigenes related to hopscotch. We could not identified the reaining components of this pathway (unpaired, STAT, JAK, and the receptor Domeless/Master of marelle (DOME/MOM)) [146, 147]. We also identified 9 unigenes related to the JNK-signaling pathway. Five unigenes were related to Kay and 4 to hemipterous.

## Conclusion

*M*. *alternatus* is known as the cancer of pine trees and is considered a devastating disease, causing serious environmental and economic losses. Vector control strategies are needed to stop and/or control the spreading of this disease [5]. In this work, we sequenced and characterized the transcriptome in the insect vector *M*. *alternatus* using Illumina sequencing. We identified a large set of genes related to putative insecticide resistance, intestinal digestive enzymes, possible future insect control targets and immune-related molecules. This study provides valuable information that may serve as key point to develop new control strategies for Pine Wilt Disease.

## Materials and Methods

### Ethics Statement

There are no specific permits for insect collection in the selected locations. The chosen locations are not privately-owned or natural protected areas. Insects used for the experiments are not considered endangered or protected species, and its collection is legal in China.

### Insects

*Pinus massoniana* (*P*. *massoniana*) trunks infested with *M*. *alternatus* were selected from trees withered during the first year in the town of GuanTou, LianJiang county and FuJian province (N 26.15046°;E 119.59261°). Trunks were cut into 1 m size with a chain saw in the open field and transported to the isolated laboratory of FuJian Agriculture and Forestry University in sealed canvas bags. Trucks were kept in a rearing cage (1.5 m x 1.0 m in length/width) with 1mm iron mesh to prevent insect scaping. Insects were maintained using an artificial diet and kept for two generations. Twenty-five whole larvae (fourth instar) were collected for RNA extraction.

### cDNA library and Illumina sequencing

Total RNA was extracted from 25 whole larvae (fourth instar) using TRIzol Reagent (Invitrogen). Extracted RNA was processed using the E.Z.N.A. ^®^ HP Total RNA Kit (OMEGA RNA, Invitrogen) to eliminate polysaccharides. RNA purity, quality and concentration were determined using Nanodrop, Qubit 2.0 and Agilent 2100 methods. Messenger RNA was extracted from 6 μg (50 ng/ul) of total RNA, using oligo (dT) magnetic beads, fragmentation buffer was added to the beads coated with mRNA and mRNA was broken randomly. mRNA was used to synthetize the first cDNA chain and then subjected to a second amplification to obtain double stranded cDNA. Double stranded cDNA was purified using AMPure XP. Finally, the cDNA library was created by PCR enrichment using HiSeq2500 high-throughput sequencing, the read length for the sequencing was PE125.

### Bioinformatic analysis

The cDNA library was sequenced by High-throughput sequencing platform to produce a large number of high quality reads based on Sequencing by Synthesis (SBS) technology. Raw data were cleaned from joint sequence and low-quality reads. Depurated reads were assembled into contigs using Trinity software. The transcript sequences were identified in each fragment collection using the De Bruijn method of graphing the sequencing read information [9]. The BLAST parameter E-value was set at 10^−5^, and the HMMER parameter E-value was set at 10^−10^. All unigenes were compared with NR [148], Swiss-Prot [149], GO [150], COG [150], KOG [151] and KEGG [152] databases using BLAST [153] software. GO database was used to determine the function of the identified transcript and to assign Gene Ontology (GO) terms. In addition, metabolic pathways were predicted using the COG and KEGG databases. Amino acid sequence of the unigenes was analyzed by HMMER [154] software and the outputs were searched in the Pfam [155] database to obtain annotated information for the unigenes.

## Supporting Information

S1 Fig Contigs length distribution. (TIF)

S1 Table Gene Ontology of *Monochamus alternatus* Hope unigenes. (XLSX)

S2 Table KEGG summary of *Monochamus alternatus* Hope transcriptome. (XLSX)

S3 Table Putative P450 genes identified in *Monochamus alternatus* Hope transcriptome. (XLSX)

S4 Table Identified genes in *Monochamus alternatus* Hope transcriptome. (XLS)
