# Supplementary figures and images for: Identification of Genes Relevant to Pesticides and Biology from Global Transcriptome Data of Monochamus alternatus Hope (Coleoptera: Cerambycidae) Larvae
Source: PLoS One. 2016 Jan 27;11(1):e0147855. doi: 10.1371/journal.pone.0147855 (PMC4729689; doi:10.1371/journal.pone.0147855)

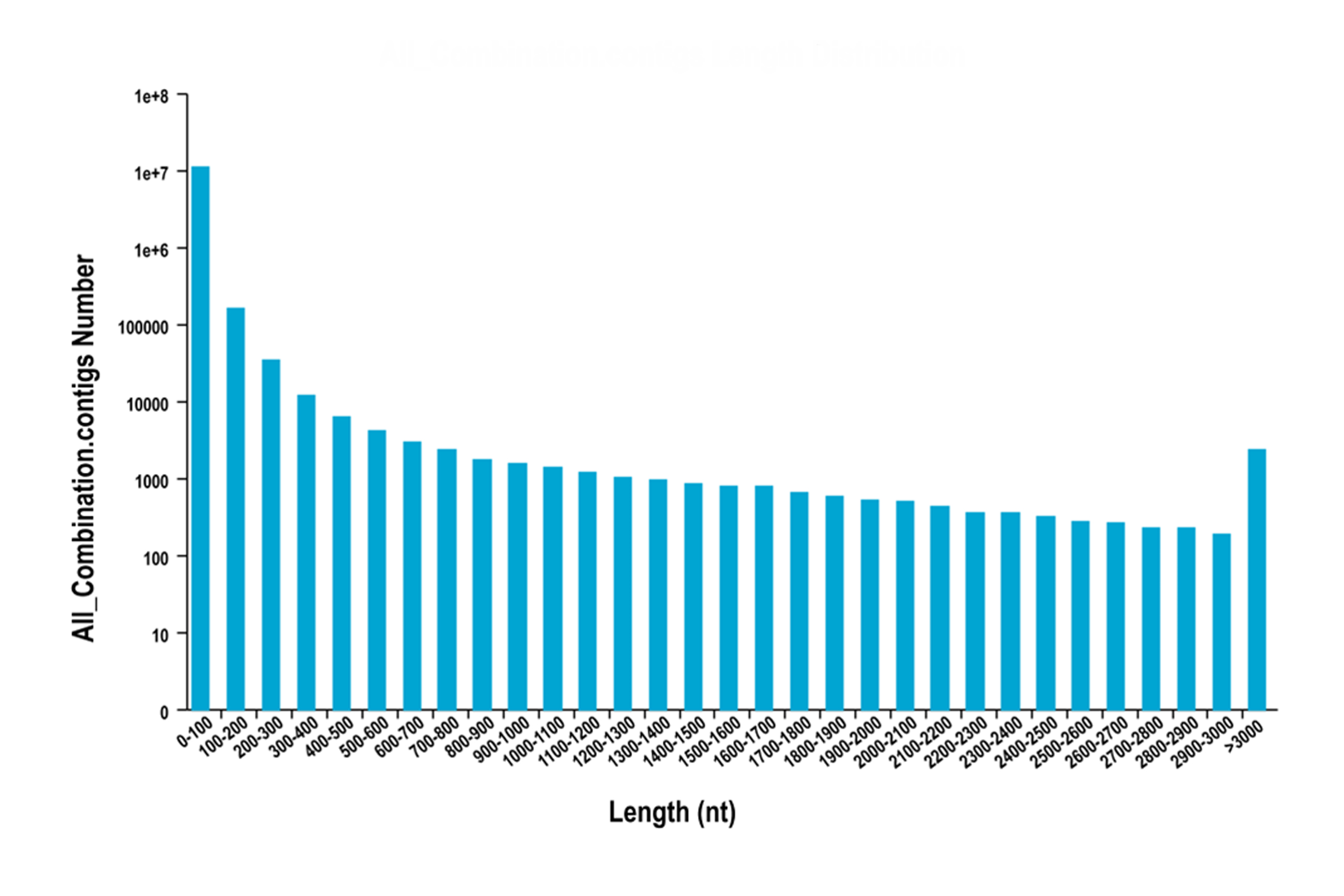

Supplement: S1 Fig — (TIF) [file pone.0147855.s001.tif]
